# RAB40C regulates RACK1 stability via the ubiquitin–proteasome system

**DOI:** 10.4155/fsoa-2018-0022

**Published:** 2018-07-02

**Authors:** Jon P Day, Ellanor Whiteley, Michael Freeley, Aideen Long, Beatrice Malacrida, Patrick Kiely, George S Baillie

**Affiliations:** 1 Institute of Cardiovascular & Medical Sciences, University of Glasgow, Glasgow G12 8QQ, UK; 2 Department of Clinical Medicine, Institute of Molecular Medicine, Trinity College, Dublin, D08 W9RT, Ireland; 3 Materials & Surface Science Institute & Health Research Institute, University of Limerick, Limerick, Ireland

**Keywords:** RAB40C, RACK1, ubiquitin–proteasome system

## Abstract

**Aim:**

RACK1 is a multifunctional scaffolding protein that is expressed in many cellular compartments, orchestrating a number of signaling processes. RACK1 acts as a signaling hub to localize active enzymes to discrete locations; therefore tight control of RACK1 is vital to cellular homeostasis. Our aim was to identify the mechanisms responsible for RACK1 turnover and show that degradation is directed by the ubiquitin proteasome system.

**Results:**

Using siRNA screening, we identified RAB40C as the ubiquitin E3 ligase responsible for ubiquitination of RACK1, and that the action of RAB40C in controlling RACK1 levels is crucial to both cancer cell growth and migration of T cells.

**Conclusion:**

Our data suggest that manipulation of RACK1 levels in this way may provide a novel strategy to explore RACK1 function.

## Lay abstract

When cells in the body grow and divide there is a coordination underpinned by intra- and inter-cell communication. This process depends on anchoring proteins that bring cell communication proteins together in the correct amounts, in the correct place, and at the right time. RACK1 is a scaffolding protein that is known to fulfill this function and as a result a balance of cellular synthesis versus controlled breakdown tightly controls its concentration in cells. This study, for the first time, demonstrates that RACK1 concentration and function is controlled by another protein called RAB40C, which ‘tags’ it for destruction by the 26S proteasome. This may have therapeutic implications for certain kinds of cancerous cell growth.

RACK1 is a highly conserved intracellular adaptor protein. The protein was named Receptor for Activated C Kinase 1 (RACK1) due to its association with the active form of PKC [1] where it was identified as both an anchor [2] and activator of the kinase [3]. Recent work has identified RACK1 as a versatile scaffold protein, integrating inputs from distinct signaling pathways to build large complexes that are required for a series of fundamental cellular activities. The seven WD-repeats of RACK1 adopt a propeller structure, where blades are arranged radially around a central axis [4] presenting multiple protein-binding sites, which facilitate interactions with specialized protein docking modules such as SH2 domains, pleckstrin homology domains, PDZ domains and C2 domains (PKCs) (reviewed in [5]).

The diverse scaffolding role of RACK1 has been shown to be integral to important processes such as neural development [6], translation [7], maintenance of cell cycle [8,9], receptor trafficking [10], circadian rhythms, cell death [11] and cell migration [12,13]. The majority of these functions are orchestrated by approximately 80 protein interaction partners of RACK1 that have been reported to date. Of key importance to the unique ability of RACK1 to coordinate signaling events in a multitude of different cellular locations is the maintenance of the scaffold's stoichiometric ratio with its interactome. Indeed aberrant RACK1 expression is reported in disease states such as Alzheimer's disease [14], Down's syndrome [15], and cancer [16], and RACK1 expression is known to decrease with age in the rat brain and human leukocytes [17]. In light of this, it is surprising that there is a lack of information on factors that control RACK1 levels in cells/tissue and the half-life of this vital anchor protein.

Ubiquitination of proteins is known to involve a multistep reaction catalyzed by three classes of enzymes, ubiquitin-activating enzymes (E1), ubiquitin-conjugating enzymes (E2) and ubiquitin-protein ligases (E3), which act to bring the E2s and substrates together. While the human genome encodes only a handful of E1 and E2 proteins, there are approximately 600 E3s which are consistent with their role of increasing specificity to the ubiquitination process by ultimately selecting substrates.

The E3s can be subdivided by homology into homologous to E6-AP C-terminus and RING types. Homologous to E6-AP C-terminus E3 ligases have a scaffolding and catalytic role in that they transiently bind to the ubiquitin via a conserved cysteine during the final transfer, whereas RING types are merely scaffolds that orientate E2s into the correct position for effective conjugation of ubiquitin to substrates. RINGs can be single polypeptides that contain the substrate binding site and E2 docking domain or can be made up from multiple proteins in a RING complex. The canonical multisubunit RING is called as SCF (SKP/Cullin/Fbox) complex comprising of a cullin-type scaffold that binds the E2-recruiting protein RBx and the SKP protein that bridges the substrate recruiting F-box [18]. The F-box is variable within this complex and therefore it is the F-box that determines substrate specificity. Many of the F-box proteins, like RACK1, contain WD repeat motifs [19] and it has been proposed that RACK1 may function as an E3 ligase itself [20]. The ubiquitin–proteasome system (UPS) is one of the best known proteolytic mechanisms for the timely destruction of signaling proteins, and it has been shown to feature prominently in many of the biological processes directed by RACK1, including the cell cycle and circadian rhythms [21].

Here we report for the first time that RACK1 has a relatively short half-life and is turned over by the UPS following conjugation of ubiquitin chains that tag it for destruction by the 26S proteasome. Using siRNA screening we have also identified a putative E3 ligase for RACK1, namely RAB40C, an atypical Rab protein and small GTPase that contains a SOCS box [22]. We show that silencing of RAB40C results in the upregulation of RACK1, which in turn can reduce proliferation and colony formation of HCT116 colon cancer cells and influence the migration of T cells.

## Materials & methods

### Cell culture & transfection

HEK 293 and HCT 116 colorectal carcinoma cell lines were purchased from ATCC (VA, USA). Cells were cultured in Dulbecco's Modified Eagle's Medium (DMEM) supplemented with 10% (v/v) fetal bovine serum, 10 mM L-glutamine and 5 mg/ml penicillin/streptomycin (all Sigma [Welwyn Garden City, UK]) in humidified air with 5% CO_2_ at 37°C. Transient transfection of HEK 293 cells were performed using PolyFect (Qiagen, Manchester, UK) as per the manufacturer's protocol. SMARTpool siRNA was purchased from Dharmacon (CO, USA). Transfection of siRNA oligonucleotides against RAB40C and a nontargeting control were incubated for 48 h using the NEON transfection system^®^ (ThermoFisher Scientific, Renfrew, UK). HEK 293 cells were treated with cycloheximide and MG132 (both Sigma) as described in the text.

Peripheral blood mononuclear cells were isolated from buffy-coat blood packs by Lymphoprep™ (STEMCELL Technologies, Cambridge, UK) density centrifugation. Monocytes were subsequently depleted by adherence to T175 cm^2^ flasks, and the nonadherent suspension cells were stimulated with 5 μg/ml phytohemagglutinin for 72 h, followed by 20 ng/ml recombinant human IL-2 for 5 days to produce activated human T-cell blasts [23]. The purity of the cells was typically >97% CD3^+^ T cells as measured by flow cytometry. The T cells were subsequently transfected with 1000 nM of nontargeting or RAB40C siRNAs using our optimized ‘two-hit’ nucleofection procedure over a 5-day period [23]. Cells were then re-suspended at 2.5 × 10^6^ cells/ml in 0.5% BSA/RPMI-1640 (Thermo FIsher Scientific) and incubated for 2 h at 37°C to deplete the cells of cytokines (i.e., recombinant IL-2) and chemokines present in the fetal bovine serum.

### Immunoprecipitation, antibodies & western blotting

Cells were lyzed in 3T3 lysis buffer (50 mM HEPES [pH 7.2], 10 mM EDTA, 100 mM NaH_2_PO_4_:2H_2_O, 1% Triton X-100) supplemented with protease inhibitor tablets (Roche, Welwyn Garden City, UK). Detergent insoluble proteins were pelleted by centrifugation at 13,000 × *g* for 10 min. Myc-RACK1 and HA-ubiquitin were immunoprecipitated from cell lysates containing 500 μg total protein and were equalized to a total volume of 500 μl. Lysates were incubated with rotation overnight at 4°C with Myc or HA beads (Sigma) before beads were washed three-times with 3T3 lysis buffer. Complexes were eluted by boiling them in SDS-loading buffer (10% SDS, 300 mM Tris-HCl pH 7.2, 0.05% bromethanol blue, 50% glycerol, 10% β-mercaptoethanol).

Western blotting of cell lysates and immunoprecipitations were performed with the NuPAGE system (Invitrogen, Renfrew, UK) following the manufacturer's instructions. Resolved proteins were transferred onto nitrocellulose membranes (Whatman, Little Chalfont, UK) in 1× transfer buffer (Invitrogen) with 20% methanol for 2 h at 25 V. Membranes were blocked in 5% Marvel milk powder/tris-buffered saline with Tween (TBST) (25 mM Tris-HCl pH 7.6, 100 mM NaCl, 0.5% Tween 20) for 1 h at room temperature, before probing with primary antibodies in 1% milk/TBST overnight at 4°C at an appropriate dilution. Membranes were washed three-times with TBST and incubated with the corresponding peroxidase secondary antibody in 1% milk/TBST for 1 h at room temperature. Enhanced chemiluminescence western blotting substrate (Thermo Scientific) was used to detect immune bands on blue x-ray film. Densitometry on film was performed using Quantity One software (BioRad Laboratories, Watford, UK). Antibodies used for western blotting and immunoprecipitation were as follows, RACK1 (Santa Cruz, CA, USA: sc-17754), tubulin (Abcam, Cambridge, UK: ab4074), HA (Abcam, UK: ab18181), ubiquitin (Santa Cruz: sc-8017, P4D1), MYC (Cell Signalling Technologies, MA, USA: mAb 2276), Actin (Abcam, UK: ab1801).All antibodies were used at dilutions recommended by the manufacturer.

### Colony formation assay

Transfected HCT116 cells were harvested with trypsin/EDTA, washed with DMEM and counted using a hemocytometer. 500 cells per well of a six-well plate were plated and incubated at 37°C in 5% CO_2_ for 8 days. Cells were then fixed in 96% ethanol for 10 min and subsequently stained with 0.05% crystal violet for 20 min. The wells were washed carefully and allowed to dry before counting. Colonies with >50 cells were scored as positive.

### Real-time proliferation analysis

The rate of HCT116 colorectal carcinoma cell proliferation was monitored in real time with the xCELLigence system (ACEA Biosciences, CA, USA) as described previously [24]. Thirty thousand transfected cells were plated in each well in DMEM. The impedance value of each well was automatically monitored by the xCELLigence system (xCELLigence RTCA DP, ACEA) for duration of 65 h and expressed as a cell index value.

### T-cell transwell migration assay

Transwell migration assays were carried out using 3 μm transwell filters (Neuroprobe ChemoTX 96-well plate migration system, Neuro Probe, MD, USA) previously coated with recombinant human ICAM-1-Fc (5 μg/ml anti-Fc antibody followed by recombinant human ICAM-1-Fc at 1 μg/ml). The bottom chambers of the transwells contained the chemokine SDF-1α (50 ng/ml). Control transwell migration assays were performed in parallel in the absence of ICAM-1 and SDF-1α. All samples were analyzed in triplicate. Cells were loaded onto the upper side of the filters and cells that had migrated to the bottom chambers were harvested after 3 h at 37°C. The cells were stained with the nuclear dye Hoeschst 33258 and quantified by High Content Image Analysis (IN Cell 1000 analyzer), which automatically counts the number of cells based on the uptake of the Hoechst dye. The percentage of cells that had migrated through the filters was determined by counting the input of cells loaded on top of the filters.

## Results

**Figure 1.**
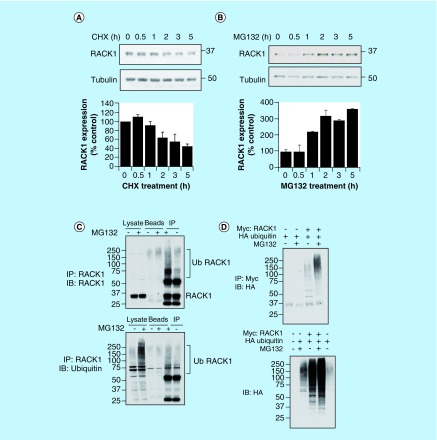
**RACK1 turnover is influenced by ubiquitin–proteasome system.** Endogenous RACK1 expression in HEK 293 cells was evaluated by western blot following a time course of **(A)** 50 μg/ml cycloheximide and **(B)** 20 μM MG132. Bar charts in lower panels represent the mean of three independent experiments. **(C)** RACK1 was immunoprecipitated from control HEK 293 cell lysate or lysate that had been extracted from cells pretreated with MG132. Immunoprecipitates were blotted for either RACK1 (upper panel) or ubiquitin (lower panel). **(D)** HEK 293 cells were transfected with HA-tagged ubiquitin and Myc-tagged RACK1 and treated with MG132. Myc-RACK1 was immunoprecipitated from cell lysates and blotted for HA-ubiquitin (upper panel). Cell lysates were blotted for HA (lower panel). HA: Human influenza hemagglutinin.

As the UPS is a highly regulated mechanism used by cells to control the local concentration of important protein signaling intermediates, we were interested to discover whether RACK1 was regulated in this manner. Treatment of HEK 293 cells with cycloheximide, an inhibitor of protein synthesis, rapidly diminished the amount of RACK1 in cellular lysates, with the scaffold exhibiting a half-life of approximately 3 h (Figure 1A).

As such rapid turnover of a protein can be facilitated by the UPS, we next treated HEK 293 cells with the proteasome inhibitor MG132. On blockade of the proteasome, the concentration of RACK1 doubled after an hour and trebled after 2 h, suggesting that turnover of the scaffold was, at least in part, conducted by the proteasome (Figure 1B). Targeted destruction of proteins is often directed by conjugation of ubiquitin chains to surface associated lysine residues by ubiquitin ligases. In an attempt to determine whether RACK1 could be modified by ubiquitin, we immunoprecipitated RACK1 from cellular lysates of HEK 293 cells and blotted for ubiquitin. The proteasome inhibitor MG132 was effective in upregulating the total amount of ubiquitinated proteins in cellular lysates and in increasing the higher molecular weight ubiquitin conjugates associated with RACK1 immunopurifications (Figure 1C, lower panel). Similarly, when RACK1 immunopurifications were blotted for RACK1, higher molecular RACK1 species were more prominent in pull-downs isolated from cellular lysates that had been prepared from cells pretreated with MG132 (Figure 1C, upper panel). These results suggest that under basal conditions, RACK1 levels are regulated by the proteasome via ubiquitin tagging, and that ubiquitinated RACK1 species accumulate when the proteasome is inhibited. To confirm this notion, we employed overexpression of HA-ubiquitin and Myc-RACK1 in HEK 293 cells. Co-expression of these proteins and subsequent immunopurification of the Myc tag showed that RACK1 ubiquitination could be forced by overexpression of HA ubiquitin and that the total amount of Myc–RACK1/HA–ubiquitin conjugates increases following MG132-induced proteasome inhibition (Figure 1D, upper panel). In agreement with this, higher molecular weight HA–ubiquitin conjugates could be seen in cellular lysates when both proteins (HA-ubiquitin and Myc-RACK1) were expressed and this could be augmented following MG132 treatment (Figure 1D, lower panel).

**Figure 2.**
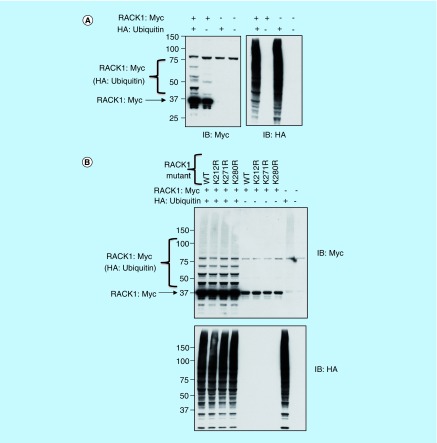
**Investigating sites of RACK1 ubiquitination.** **(A)** HEK 293 cells were transfected with Myc-tagged RACK1 and HA-tagged ubiquitin. Cell lysates were blotted for Myc (left panel) and HA (right panel). **(B)** HEK 293 cells were transfected with HA-tagged ubiquitin and both wild-type and mutated (as indicated) Myc-tagged RACK1. Cell lysates were blotted for Myc (upper panel) or HA (lower panel).

Unlike certain post-translational modifications (e.g., phosphorylation, SUMOylation) that are characterized by well-defined and identifiable amino acid motifs, ubiquitination can occur on any available lysine residue and is often determined by the spatial relationship between the E2/E3 ligase complex and protein substrate. Although RACK1 has 17 lysines, we utilized the ubiquitination site predictor ‘UbPred’ (www.ubpred.org) to evaluate the probability of each lysine being modified by ubiquitin [25]. Only three of the lysines were predicted to be possible sites of ubiquitination K212 (medium confidence), K271 (low confidence) and K280 (medium confidence), hence we mutated these to arginine. When cellular lysates were probed using the Myc tag following overexpression of Myc-RACK1, a number of molecular weight species were isolated that corresponded to different RACK1 ubiquitination states (Figure 2A, upper panel lane 2).

As expected, the number and intensity of the RACK1–ubiquitin conjugates increased when RACK1 was co-expressed in HEK 293 cells with HA-ubiquitin (Figure 2A, upper panel lane 1). Using mutants of RACK1 where the predicted ubiquitin sites were substituted with arginine (K212R, K271R, K280R), we endeavored to attenuate the conjugation of ubiquitin while maintaining the net charge of the protein sequence. Unfortunately, although transfection of HA-ubiquitin caused a robust increase in ubiquitinated proteins (Figure 2B, lower panel), in disagreement with the prediction software, no mutant RACK1 constructs exhibited diminution in the intensity or the number of ubiquitinated RACK1 conjugates (Figure 2B, upper panel), hence it seems unlikely that any of these lysines represent sites for ubiquitin modification on RACK1.

**Figure 3.**
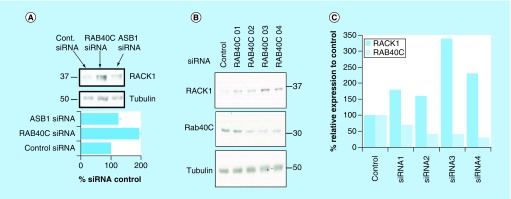
**Identifying a putative ubiquitin E3 ligase for RACK1.** We utilized a SMARTpool siRNA library against all known human F-box proteins in HEK 293 cells to systematically silence this class of E3 ligase. **(A)** Cellular lysates from cells transfected with each SMARTpool and siRNA controls were blotted for RACK1. **(B)** Individual siRNA oligonucleotides used in the RAB40C smart pool were tested for their ability to silence RAB40C and increase RACK1 expression. **(C)** Protein levels of RAB40C and RACK1 normalized to tubulin were evaluated using densitometry (n = 1).

Proteins which are targeted for proteolysis by the 26S proteasome following ubiquitin modification are selected specifically by their ability to bind one or more of the around 600 E3 ligases that orientate the E2 ubiquitin-conjugating enzyme into the correct position to covalently link ubiquitin to surface-associated lysine residues. The large number of E3 ligases means that each protein from this family has a small group of substrates, which it often selects by way of a distinct E3 docking motif found on each of the substrates. In a bid to discover which E3 ligase facilitates ubiquitination of RACK1, we utilized a Dharmacon SMARTpool siRNA library (which contained four specific siRNA oligonucleotides targeted to different parts of the same protein) against all known human F-box proteins in HEK 293 cells (see Methods). Analysis of the RACK1 expression in cells that had individual F-box proteins silenced showed that only one E3 enzyme specifically and robustly increased protein levels of RACK1 when it was knocked down, RAB40C (Figure 3A). Silencing of RAB40C (but no other E3 ligase, e.g., ASB1, a randomly selected F-Box) (Figure 3A) resulted in doubling of RACK1 protein levels. Analysis of the individual siRNA constructs showed that, when utilized individually, all four worked to silence RAB40C to some degree (Figure 3B), with the most effective knockdown (RAB40C 03 and RAB40C 04) producing the largest increase in RACK1 levels (Figure 3C).

**Figure 4.**
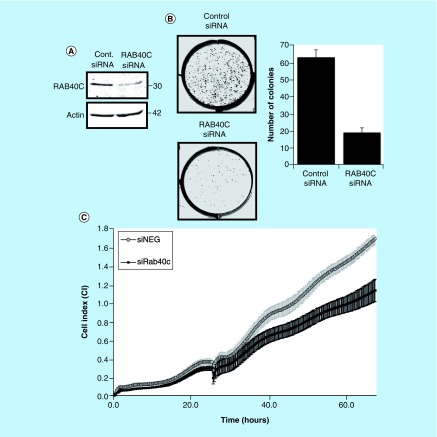
**Effect of knockdown of RAB40C on colon cancer cells.** **(A)** Representative western blot showing RAB40C knockdown (10 nM) in HCT116 (n = 3). **(B)** Representative image demonstrating the difference in cells ability to form colonies when RAB40C is knocked down. Bar graph shows the different number of colonies scored as positive (>50 cells) after 8 days (in six different wells repeated in two individual experiments). **(C)** Representative graph of cell proliferation over a period of 65 h analyzed in real time on the xCELLigence system. Standard deviation calculated on the average of eight different wells.

Next, we set out to show that the control of RACK1 levels by RAB40C can have functional consequences in the transformation and proliferation of cancer cells, and in the migration of T cells. Changes in RACK1 expression have been recorded in various cancers including colon [8], gastric [26], lung [27] and breast cancer [16]. Using the colon cancer cell line HCT116, we successfully silenced RAB40C using siRNA (Figure 4A), which significantly reduced colony formation (Figure 4B). Using the xCELLigence platform, which has been extensively used to monitor cancer cells in real time [28], we were able to use real-time, noninvasive monitoring of cell growth to show that RAB40C knockdown also significantly reduced the proliferation of HCT116 cells (Figure 4C).

**Figure 5.**
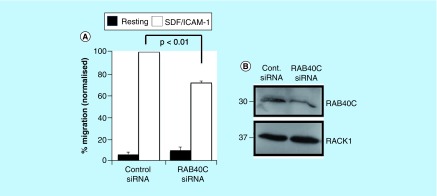
**Silencing of RAB40C expression in T cells perturbs migration.** **(A)** Primary-activated human T cells were transfected with control siRNAs or siRNAs targeting RAB40C. The cells were subsequently loaded onto transwell filters coated with ICAM-1, and migration toward the chemokine SDF-1 in the bottom chamber was quantified. The numbers of cells migrating toward SDF/ICAM-1 in the control siRNA samples was set to 100%. The data in **(A)** were compiled from three independent experiments. (*) signifies statistical significance as p < 0.05 using a student's *t*-test. **(B)** Representative western blot demonstrating silencing of gene expression of RAB40C and upregulation of RACK1 protein.

As RACK1 expression is known to be important for integrin-dependent cell migration [29] and it had previously been shown that knockdown of RACK1 expression resulted in an increase in lymphocyte migration toward the chemokine SDF-1 [30] we tested whether manipulation of RACK1 protein levels via RAB40C silencing would alter the ability of T cells to migrate toward a chemotactic stimulus (Figure 5). As seen in HEK 293 cells (Figure 3), RAB40C silencing produced an increase in RACK1 protein levels in HCT116 cells (Figure 5B). Furthermore, we investigated whether the increase in RACK1 expression associated with attenuated RAB40C expression would result in the inhibition of chemokine-stimulated migration in T cells (Figure 5A). Clearly, in the absence of ICAM-1 and SDF-1, the motility of these cells was very low in both control and RAB40C-depleted cells; a significant decrease in the ability of T cells to migrate through ICAM-1-coated filters toward SDF-1 was observed following RAB40C silencing. Our results are in agreement with those from an earlier report that demonstrated in Jurkat and neutrophil-like differentiated HL60 cells [30], that upregulation of RACK1 acts as a negative regulator of chemoattractant-directed immune cell migration.

Both of the functional examples highlighted above, in different physiological settings, highlight the importance of RAB40C in maintaining RACK1 expression at the correct level.

## Discussion

Scaffolding proteins orchestrate cellular signaling by acting as control centers for the integration of information and dissemination of specific environmental cues. Inherent in the definition of a scaffolding protein is the ability to function as a ‘hub’ by localizing signaling molecules and components to specific compartments of the cell. It is clear that the stoichiometry of the interactions directed by anchors such as RACK1 are very tightly controlled and subtle changes in expression or availability of scaffolding proteins can have dramatic consequences in the cell, leading to disease initiation and progression. RACK1 expression, for example, is known to decrease with age [17] and this correlates with plasma levels of dehydroepiandrosterone (DHEA) [31]. Direct control of RACK1 expression by DHEA is regulated through enhancer and silencing elements within a small, defined region of the GNB2L1 gene, and DHEA treatment in the elderly may promote recovery of RACK1 expression. Apart from this observation, there is no information available on the cellular mechanisms used to maintain RACK1 at the desired levels in different cell types, or on the half-life of the protein. We have shown that RACK1 is turned over relatively quickly and that half-life depends on the action of the UPS. Such an observation is interesting considering previous reports that RACK1 is an integral part of Cullin 2-based ubiquitin E3 ligase complexes that degrade HIF-1 α [32] and BimEL [33]. Although RACK1 has been shown to be modified by phosphorylation [34,35] and SUMOylation [36], our report is the first to show that RACK1 itself is a substrate for ubiquitin, a result that is in agreement with observations from a recent paper that found the scaffold in complex with the deubiquitinating enzyme otubain-1 [37].

As can be expected from RACK1's important role in the cell cycle, there are many reports of altered RACK1 expression in variety of cancers (reviewed in [38]). In the context of colon cancer, RACK1 expression is shown to inhibit cancer cell growth by delaying cell-cycle progression and promoting apoptosis [8]. This dual process relies on two distinct mechanisms [39], the first of these being the direct suppression of Src activity by RACK1, leading to attenuation of mitotic exit and entrapment of cancer cells in G2/M phase. The second mechanism is underpinned by blockade of the Akt cell survival signaling pathway. In light of this, we were curious as to whether we could replicate this via silencing of RAB40C in a colon cancer cell line. In agreement with the studies outlined above, RAB40C silencing retarded colon cancer cell growth. It should be noted that the involvement of RAB40C in gastric cancer has previously been investigated [40]; however, this is the first report in which the enzyme also has a role in colon cancer.

The functional roles of the small GTPase RAB40C are varied and include biogenesis of lipids [41] and vesicle transport [42]. The ubiquitin function of RAB40C proteins stems from its ability to interact with Cullin5 to form an E3 ubiquitin ligase complex which can influence Wnt signaling via modification of Rap2 GTPase [39]. More recently, it has been shown that the RAB40C recruited ubiquitin ligase activity can also influence the cellular concentration of Varp (VPS9-ankyrin repeat protein), an activator of the small GTPase Rab21 that functions as a crucial signaling intermediate in the mammalian pigmentation process [43]. In agreement with our data included here for RACK1 (Figure 3), RAB40C silencing in melanocytes resulted in a significant increase in the cellular concentration of Varp [43]. As ubiquitination of Varp by the Cullin5/RAB40C complex depends on a protein–protein interaction between Varp and RAB40C, mediated by the ankyrin repeat 2 (ANKR2) on Varp, we were interested to note a report that described an interaction between RACK1 and Varp [44]. Intriguingly, RACK1 and RAB40C appear to compete for Varp ANKR2, making binding of these proteins mutually exclusive. Overexpression of RACK1 serves to inhibit the interaction between Varp and RAB40C, protecting the former from degradation in mouse melanocytes. RACK1 is also known to protect another ubiquitin substrate c-Jun [45] by a similar mechanism, in this circumstance blocking c-Jun's binding to the E3 ligase FBW7. These examples appear to contradict many other occasions where RACK1 itself promotes ubiquitination and degradation of target proteins such as HIF-1α [32], P63 [46], lectin receptor CLEC-2 [47], the proapototic FEM-1 [48] and the multidrug resistance protein MDR3 [49].

## Conclusion

The protein concentration of RACK1 is fine-tuned by the UPS to maintain the correct stoichiometry of signalling complexes orchestrated by the scaffold. When RACK1 levels are depleted, aberrant signalling can affect important cellular processes such as cancer cell growth and T cell migration.

## Future perspective

We speculate that aberrant turnover of RACK1 has negative cellular repercussions through dysregulation of the cell cycle, signaling complexes and protein stability. Manipulation of the UPS machinery described here may provide a novel approach to influence the functions of RACK1 in cell migration, circadian rhythm, development and disease. A novel therapeutic approach for some cancer types could be developed from proto-type protein–protein interaction disruptors that selectively disrupt the RAB40C–RACK1 complex.

**Executive summary**

- RACK1 has a short half-life and is turned over by the ubiquitin–proteasome system.
- RAB40C is an E3 ubiquitin ligase for RACK1.
- RAB40C control of RACK1 protein levels is important for proliferation of colon cancer cell line HCT116 and chemoattractant-directed immune cell migration.
- The RACK1–RAB40C complex may represent a novel therapeutic target.
